# Effectiveness of aerobic exercise training in patients with obstructive sleep apnea: a systematic review and meta-analysis

**DOI:** 10.1007/s00405-025-09436-3

**Published:** 2025-05-06

**Authors:** Mrudula Pawar, Prem Venkatesan, Satyanarayana Mysore, Guruprasad Bhat

**Affiliations:** 1 Department of Physiotherapy, Manipal College of Health Professions, Manipal Academy of Higher Education, Manipal, Karnataka India; 2 Department of Pulmonology, Manipal Hospital, HAL Airport Road, Bangalore, Karnataka 560017 India

**Keywords:** Endurance exercise, Apnea hypopnea index, Sleep disorders, Obstructive sleep apnea

## Abstract

**Purpose:**

Obstructive sleep apnea (OSA), a sleep-related disorder, reports significant clinical consequences, apart from its socioeconomic burden globally. Among the physiotherapeutic treatment options, exercise training is primarily preferred for these patients. In the current systematic review and meta-analysis, we hypothesize that aerobic exercise training could be beneficial in reducing the severity of OSA.

**Methods:**

A thorough literature search was carried out from Scopus, PubMed, CINAHL, Cochrane, and Embase databases following the PRISMA guidelines, and eight studies were included. The primary outcome was the apnea hypopnea index (AHI) and secondary outcomes were maximal oxygen consumption, oxygen desaturation index, mean oxygen saturation during sleep, Epworth sleepiness scale, body mass index, and neck circumference. RevMan version 5.4.1 was utilized for analysis.

**Results:**

Meta-analysis involved seven studies that showed that aerobic training significantly improved the AHI with a mean difference of -5.24 and an overall effect of *p* < 0.00001; and VO_2max_ with a mean difference of 5.84 and an overall effect of *p* = 0.03. The other secondary outcomes reported improvement but were not significant.

**Conclusion:**

The current review concludes that there is supporting evidence for the beneficial effects of aerobic exercise training in reducing the severity of obstructive sleep apnea.

**Prospero registration:**

CRD42023453316.

## Introduction

Obstructive sleep apnea (OSA) is a highly prevalent sleep disorder with nearly one billion of the population affected globally [1]. It is distinctively characterized by the presence of apnea and hypopnea episodes in sleep, as a result of the collapsible oropharyngeal airway among these patients [2]. The most common symptoms linked to this disorder are frequent nocturnal awakenings leading to sleep fragmentation and deteriorated sleep quality, loud snoring, choking and gasping in sleep, excessive daytime sleepiness, and abnormal breathing pattern during sleep [2, 3]. OSA is associated with various co-morbidities such as obesity, diabetes mellitus, hypertension, dyslipidemia, renal dysfunction, depression, and increased risk of other cardiovascular and metabolic disorders [4]. Amongst these, obesity is most closely associated to OSA severity. A study by Zou J et al. [5] reported that visceral adiposity has a significant association with OSA. Moreover, evidences suggest that the worsened OSA severity is a consequence of increased airway collapsibility and loop gain among obese patients [6, 7].

In the past few decades, OSA has become an area of key focus due to its clinical consequences in addition to its socioeconomic burden [8]. As a result of which, different forms of treatment options are researched to manage these patients. However, continuous positive airway pressure (CPAP) is considered the gold standard treatment for OSA [9]. Besides this, mandibular advancement devices, rapid maxillary expansion, hypoglossal nerve stimulation, and surgically uvulopalatopharyngoplasty are the options for those with poor compliance to CPAP due to its side effects [9]. However, Sands et al. [7] suggested that, these treatment options may subject to residual sleep apnea as they address the anatomical mechanisms particularly.

The physiotherapeutic management of OSA includes breathing exercises, respiratory muscle training, oropharyngeal exercises, and exercise training programs for the rehabilitation of these patients. Studies have been conducted to evaluate the effectiveness of these techniques in addressing issues such as excessive daytime sleepiness, sleep quality, and the severity of OSA. The outcomes of these trials have consistently shown positive results [10–13].

In the current standpoint, different forms of exercise training involving aerobic exercises and resisted exercises, are emphasized as an adjunct to the traditional CPAP treatment for OSA [14]. The recent systematic reviews and meta-analysis have reported the effect of combined aerobic exercise with resistance training and other exercise trainings [14–17]. A systematic review on adult population by Ismail I et al. [18], concluded that the independent aerobic exercise training is effective for reducing the visceral fat among individuals when compared to the resistance exercise and combined aerobic and resistance exercise training. However, aerobic exercise training alone for treating OSA patients has not yet been reviewed. The study aims to review and synthesize the evidence on the effectiveness of aerobic exercise training on the apnea hypopnea index (AHI) in patients with obstructive sleep apnea.

## Methods

A thorough and methodical literature review was put into execution to evaluate the effects of aerobic exercise training in OSA patients. “The preferred reporting items for systematic reviews and meta-analyses (PRISMA)” guidelines were followed for the study [19].

### Eligibility criteria

Published RCTs with patients diagnosed with mild, moderate, or severe OSA; Studies with aerobic training used alone or as a part of the treatment; Control or other treatment methods present as the comparator group; Human studies; Studies in English language were included. The studies using resistance training alongwith aerobic exercises in the training protocol were excluded.

### Outcomes

The primary outcome measure was AHI and the secondary outcome measures were maximal oxygen consumption (VO_2max_), oxygen desaturation index (ODI), mean oxygen saturation during sleep (Mean SpO_2_), Epworth sleepiness scale (ESS), body mass index (BMI) and neck circumference (NC).

### Information sources and search strategy

Scopus, PubMed, Embase, Cochrane and CINAHL databases were used to perform literature search with articles from 2003 to 2023. The search strategy was (“Aerobic exercise” OR “aerobic exercise training” OR “aerobic training” OR “endurance exercise” OR “endurance training”) AND (“Obstructive Sleep Apnea” OR “Obstructive Sleep Apnea Syndrome” OR “OSA” OR “OSAHS” OR “Sleep Apnea Syndrome” OR “Sleep Apnea Hypopnea Syndrome” OR “Upper Airway Resistance Sleep Apnea Syndrome”). The Medical Subject Headings (MeSH) terms used were (“Exercise“[Mesh]) AND “Sleep Apnea, Obstructive“[Mesh]. Also, the literatures were searched manually by snowballing.

### Study screening and selection

After the literature search, duplicates were removed using the Mendeley reference manager. Two independent reviewers screened the title and the abstracts of the identified studies. Full text of the included studies was assembled and their references were also researched for inclusion according to the eligibility criteria. Any discrepancies during the research selection and data extraction processes were settled through discussion with the third author.

### Data collection

The extracted data included first author, study design, study setting, publication year, and other study characteristics like participants, intervention, comparison group, outcomes, and results. The mean, standard deviation, and effect size were gathered for each study. Missing data was collected by contacting the respective authors of the studies.

### Risk of bias assessment

The two reviewers separately assessed the risk of bias of individual studies using the Cochrane Risk of Bias assessment tool [20]. The six domains of the tool consisted of random sequence generation, allocation concealment, selective reporting, blinding of participants and assessor, incomplete outcome data, and other bias. Each characteristic was rated as low risk, high risk, or unclear risk based on the information available for each study [20]. The Review Manager software (Rev Man version 5.4.1) was used for this purpose.

### Study quality assessment

The PEDro Scale was used to analyze the quality of the included studies. The scale consists of 11 criteria to assess the quality of the study. It can be scored from 0 to 11. The studies with scores more than 6 are considered to be having moderate to high quality [21, 22].

### Data analysis and synthesis of results

The studies with data presented in the form of mean ± standard deviation were included in the meta-analysis. Authors of the respective studies were contacted in case of missing data or additional unpublished data. The primary and secondary outcomes were analyzed and depicted as forest plots. The RevMan version 5.4.1 was used for meta-analysis. The fixed effect model was employed to calculate the overall effect for the included studies in quantitative analysis. I^2^ statistics were used to check heterogeneity among these studies for each outcome. Moderate, substantial, and considerable heterogeneity were indicated by an I^2^value greater than 25%, 50%, and 75%, respectively [23]. To estimate the overall effect of the outcomes at 95% confidence intervals (CI), mean differences (MDs) were calculated. The three categories of overall effect size namely, small (0.2), medium (0.5), and large (0.8) were used for analysis [24].

## Results

### Study selection

**Fig. 1:**
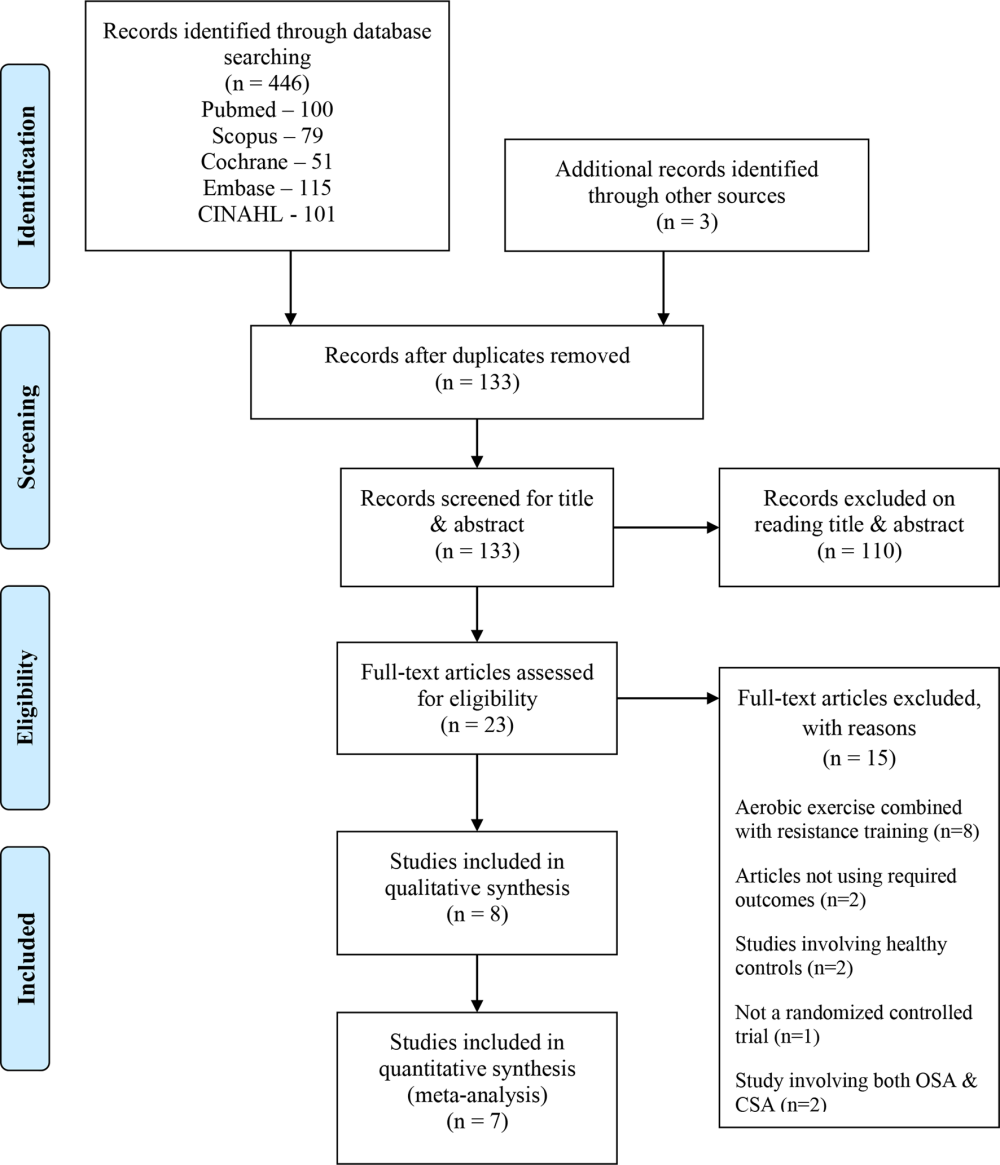
PRISMA chart

Total 446 studies were identified through searching the databases. Additionally, three studies were included by snowballing. After removing the duplicates, 133 studies were screened for title and abstract. Consequently, based on our inclusion and exclusion criteria, 110 studies were removed after reading the title and abstract and the remaining 23 studies were found to be eligible for full text reading. From the 23 studies, 15 studies were excluded due to the following reasons: aerobic exercise combined with resistance training = eight [25–32], articles not using the required outcomes = two [33, 34], studies involving healthy controls = two [35, 36], not RCT study design = one [37], and studies involving both OSA & central sleep apnea (CSA) = two [38, 39]. Finally, eight studies were included for the systematic review [40–47]. The screening of the studies is demonstrated through the PRISMA flow chart in Fig. 1.

### Study characteristics

A total of 594 participants were included through eight eligible RCTs. The aerobic exercises were combined with behavioural interventions in two studies [41, 42] and with breathing exercises in one study [46]. The frequency of exercises ranged from two days per week to seven days per week with a duration of approximately 45 to 60 min per session. The intensity of exercise in the treatment programs varied from moderate to high. Among these, two studies specifically involved high-intensity interval training for OSA patients [44, 45]. The mode of aerobic exercise in all these studies varied from ground walking to treadmill walking and bicycle ergometer. In one study CPAP was given to the controls [40]; lifestyle changes and patient education were provided to the controls in three studies [41–43]; in three studies, no treatment or continuation of the previous lifestyle was recommended to the controls [44, 46, 47]; and another study used stretching interventions in the control group [45].The characteristics and aerobic exercise protocols of the included studies are depicted in Tables 1 and 2 respectively.

**Table 1:** Characteristics of included studies

Author & Year	Place of study	Sample size (*N*)	Age (years) Mean ± SD	BMI (kg/m^2^) Mean ± SD	AHI (events/hour) Mean ± SD
Ackel-D’ Elia et al., 2011	Brazil	32 Ex: 13 Con: 19	Ex: 48.4 ± 9.2 Con: 49.5 ± 7.7	Ex: 28.0 ± 3.1 Con: 28.5 ± 2.2	Ex: 40.5 ± 22.9 Con: 42.3 ± 21.6
Carneiro-Barrera et al., 2022	Spain	89 Ex: 40 Con: 49	Ex: 52.6 ± 7.1 Con: 55.3 ± 8.5	Ex: 35.0 ± 6.0 Con: 33.9 ± 4.8	Ex: 41.6 ± 23.5 Con: 41.1 ± 21.3
Foster et al., 2009	United States	264 Ex: 125 Con: 139	Ex: 61.2 ± 6.6 Con: 61.3 ± 6.4	Ex: 36.8 ± 5.8 Con: 36.5 ± 5.7	Ex: 22.9 ± 18.0 Con: 23.5 ± 15.0
Jurado-Garcia et al., 2020	Spain	58 Ex: 34 Con: 34	Ex: 52 ± 6.6 Con: 50 ± 9.5	Ex: 32 ± 5.2 Con: 32 ± 4.3	Ex: 29 ± 19.7 Con: 27 ± 10.4
Karlsen et al., 2017	Norway	28 Ex: 13 Con: 15	Ex: 52.5 ± 7.4 Con: 49.9 ± 9.7	Ex: 38.5 ± 7.0 Con: 37.7 ± 4.8	Ex: 31.4 ± 21.7 Con: 50.3 ± 25.5
Lins-Filho et al., 2023	Brazil	36 Ex: 17 Con: 19	Ex: 53.2 ± 9.9 Con: 55.1 ± 9.8	Ex: 34.5 ± 6.6 Con: 33.8 ± 5.0	Ex: 35.6 ± 3.9 Con: 49.3 ± 6.2
Sengul et al., 2011	Turkey	20 Ex: 10 Con: 10	Ex: 54.40 ± 6.57 Con: 48.0 ± 7.49	Ex: 29.79 ± 2.66 Con: 28.42 ± 5.42	Ex: 15.19 ± 5.43 Con: 17.92 ± 6.45
Yang et al., 2018	China	67 Ex: 32 Con: 35	Ex: 46.3 ± 6.4 Con: 48.6 ± 7.2	Ex: 27.6 ± 4.7 Con: 27.1 ± 3.5	Ex: 20.2 ± 7.5 Con: 19.5 ± 6.1

Ex – Aerobic exercise group; Con – Control group; BMI – Body mass index; AHI – Apnea hypopnea index

**Table 2:** Aerobic exercise protocols of the included studies

Author & Year	Exercise program in aerobic exercise group					Control group treatment
	Intervention	Frequency	Intensity	Time	Type	
Ackel-D’ Elia et al., 2011	Aerobic Exercise + CPAP	3 times/week for 8 weeks	85% of Anaerobic Threshold	60 min	Treadmill walking or Running	CPAP for 8 weeks
Carneiro-Barrera et al., 2022	Nutritional behavior change, moderate aerobic exercise, smoking cessation, alcohol intake avoidance, and sleep hygiene	Daily for 8 weeks (Supervised once a week)	55–65% of HRR	60 min	Walking	General advise on weightloss and lifestyle change
Foster et al., 2009	Behaviouralweightloss program + moderate intensity aerobic exercise	7 days/ week for 1 year	moderate intensity	175 min/week	brisk walking	Diabetes support and education
Jurado-Garcia et al., 2020	Graduated Walking Program	5 times/week for 24 weeks	Borg Scale for Perceived Exertion − 1–4 weeks: 9–11 for warm up and cool down,11–14 for training; 5–24 weeks: 9–11 for warm up and cool down, 12–15 for training	1–2 weeks: 5 min warm up, 20 min training, 5 min cool down; 3–4 weeks: 5 min warm up, 30 min training, 5 min cool down; 5–24 weeks: 5 min warm up, 45 min training, 5 min cooldown	Walking	General therapeutic measures and regular physical activity recommended
Karlsen et al., 2017	Supervised High Intensity Interval Training	2 times/week for 12 weeks	warm up: 70% of HR_max,_ Exercise training: 90–95% of HRmax, After last interval: 70% of HR_max_	10 min warm up, 4*4 min exercise training, 3 min at last	Treadmill walking or Running	Continue normal lifestyle
Lins-Filho et al., 2023	High Intensity Interval Training	3 times/week for 12 weeks	warm-up and cool-down at ~ 40% of HR_max_, 5 cycles of 4 min walking or running between 90% and 95% of maximum heart rate (HR_max_) interspersed by 3 min of walking at 50–55% of HR_max_	4 min warmup, 35 min exercise, 4 min cool down	Treadmill walking or Running	Stretching activity + adviced unsupervised 30 min physical activity
Sengul et al., 2011	Aerobic exercise and breathing exercise	3 times/week for 12 weeks	60–70% of VO_2max_	45–60 min	Bicycle Ergometer & Treadmill	No treatment
Yang et al., 2018	Supervised Exercise Training	3 times/week for 12 weeks	At VO_2_ (Anaerobic Threshold)	15 min warm up, 15 min cool down, 30 min exercise	Bicycle Ergometer	Maintained previous lifestyle

### Study quality

The quality of the studies assessed through PEDro is shown in Table 3. Eligibility criteria and randomized allocation were followed by all the included studies while most of the studies lacked concealed allocation of participants. Moreover, information about the blinding of subjects and therapists was missing in the majority of the studies. The total PEDro scores of seven studies out of the eight were moderately high (more than or equal to 6).

**Table 3:** PEDro score

	Ackel D Elia et al., 2011	Carneiro – Barrera et al., 2022	Foster et al., 2009	Jurado – Garcia et al., 2020	Karlsen et al., 2017	Lins – Filho et al., 2023	Sengul et al., 2011	Yang et al., 2018
Eligibility	1	1	1	1	1	1	1	1
Random allocation	1	1	1	1	1	1	1	1
Concealed allocation	0	0	0	1	0	1	0	0
Baseline comparability	1	1	1	1	0	1	1	1
Blinded subjects	0	0	0	1	0	0	0	0
Blinded therapists	0	0	0	1	0	0	0	1
Blinded assessors	0	1	1	1	1	1	0	1
Outcomes for > 85%	0	0	1	1	1	1	1	1
Intention-to-treat analysis	1	1	1	1	1	1	1	1
Between-group comparisons	0	1	1	0	0	1	1	1
Point and variability measures	1	1	1	1	1	1	1	1
Total	5	7	8	10	6	9	7	9

1 = yes, 0 = no

### Risk of bias

**Fig. 2:**
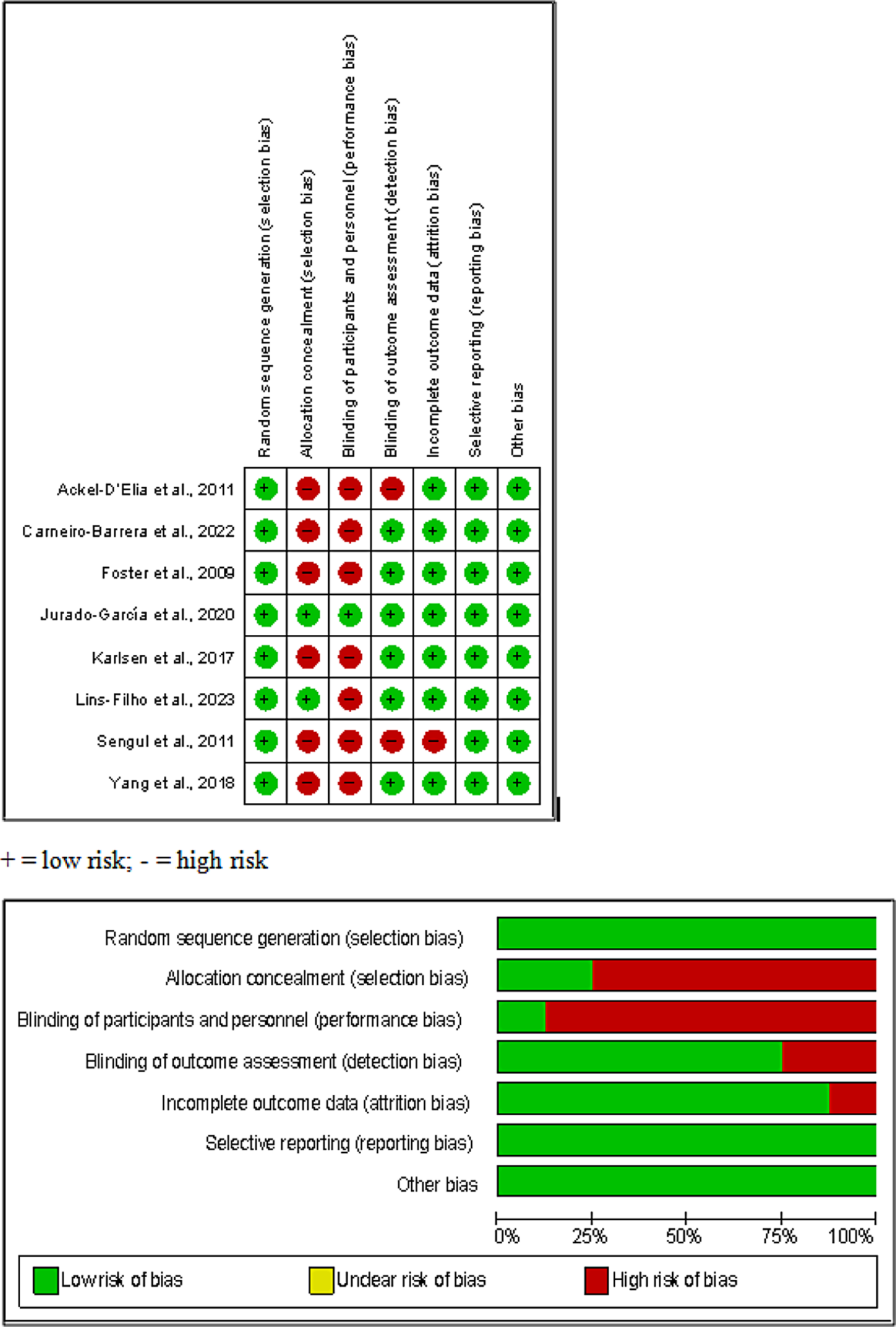
Risk of bias summary

The risk of bias of individual studies and across all the included studies is depicted in Fig. 2. Among the eight studies, one study showed a low risk of bias across all the domains of Cochrane ROB assessment [43]. A potential selection bias can be reported due to the absence of allocation concealment. A high risk of performance bias was present in the majority of the other studies. Contrarily, low risk was found for detection, attrition, and reporting bias across the studies. Two studies showed a high risk of bias overall [40, 46].

### Synthesis of results of included studies

#### Apnea hypopnea index (AHI)

**Fig. 3:**
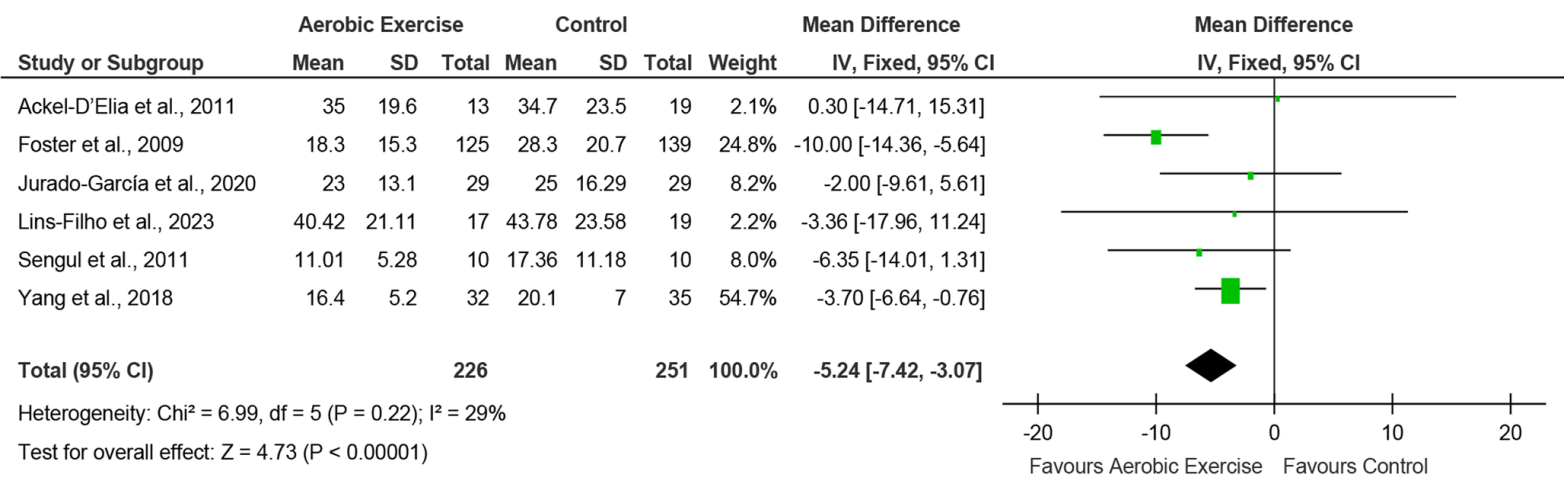
Forest plot for comparison of AHI

The meta-analysis for AHI outcome was done for six studies with a total of 226 participants in the aerobic exercise group and 251 participants in the control group [40, 42, 43, 45–47]. The other two studies were not eligible for meta-analysis as AHI was expressed as mean difference and confidence interval in one study [41] and in another study, the post-intervention values were missing for AHI outcome [44]. The results showed moderate heterogeneity among the studies (I^2^ = 29%). The effect of aerobic exercise on AHI was significant with MD -5.24 and 95% CI (-7.42, -3.07) and an overall effect of *p* < 0.00001 with a large effect size (4.73). Hence the forest plot (Fig. 3) indicated that the results favoured the aerobic exercise group.

#### Oxygen desaturation index (ODI)

**Fig. 4:**
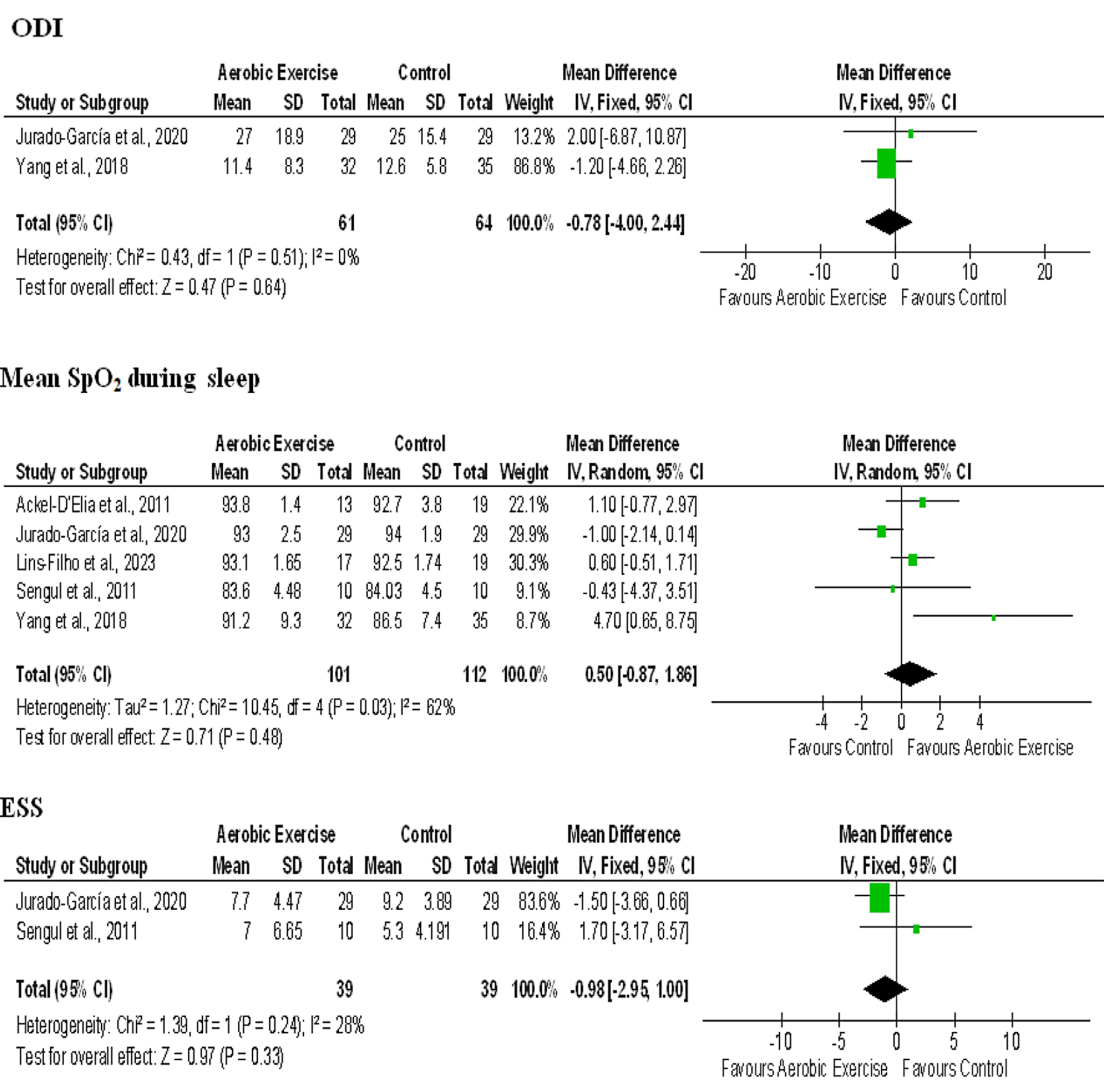
Forest plot for comparison of ODI, Mean SpO_2_ during sleep and ESS

The mean and standard deviation values of ODI outcome were available for two studies with a total of 61 participants in the aerobic exercise group and 64 participants in the control group [43, 47]. The analysis for ODI showed no heterogeneity among the studies (I^2^ = 0%). The MD was − 0.78 with CI (-4.00, 2.44) which resulted in an overall effect of *p* = 0.64 with a medium effect size (0.47), thus showing a non-significant effect (Fig. 4).

#### Mean SpO_2_ during sleep

The mean SPO2 during sleep values of five studies were available for meta-analysis [40, 43, 45–47]. These studies showed substantial heterogeneity with I^2^ = 62%. Thus random effects model was utilized to analyze the results. For mean SPO2, the MD was 0.50 with CI (-0.87, 1.86) and it resulted in an overall effect of *p* = 0.48 with a medium effect size (0.71). This denotes that the effect of aerobic exercise was not significant on Mean SpO_2_, although the values improved (Fig. 4).

#### Epworth sleepiness scale (ESS)

The mean and SD values for ESS were available for two studies with 39 participants in each group [43, 46]. These studies showed moderate heterogeneity with I^2^ = 28%. The mean difference was − 0.98 and CI (-2.95, 1.00) which gave an overall effect of *p* = 0.48 which was not significant but showed a large effect size (0.97). Although the ESS values supported aerobic exercise in one study [43], they showed contradictory results in another study [46] (Fig. 4).

#### Body mass index (BMI)

**Fig. 5:**
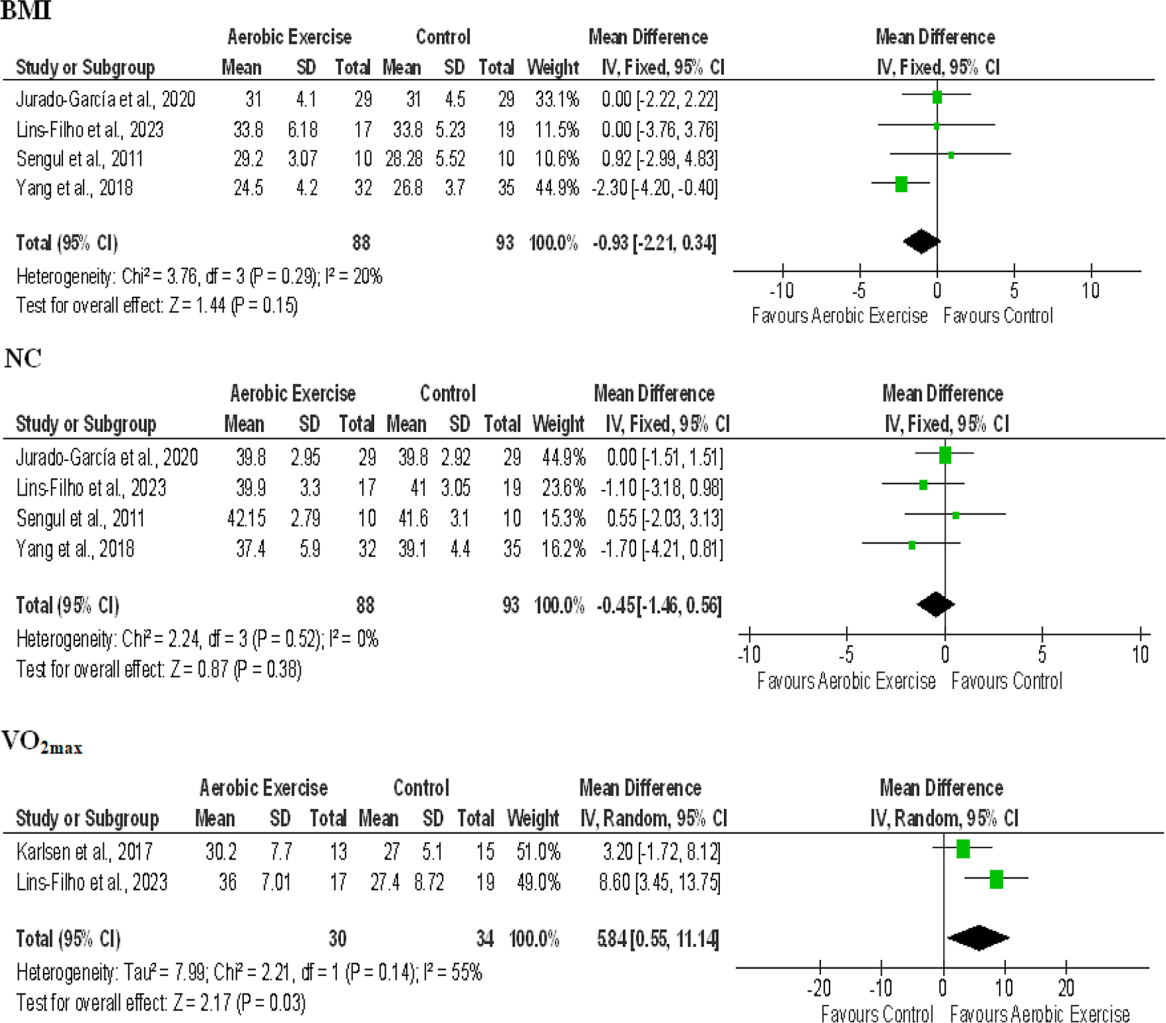
Forest plot for comparison of BMI, NC and VO_2max_

Among the included studies, four studies reported BMI values pre and post intervention that involved a total of 88 participants in the aerobic exercise group and 93 participants in the control group [43, 45–47]. There was comparatively moderate heterogeneity among the studies with I^2^ = 20%. The MD and CI were − 0.93 and (-2.21, 0.34) respectively which gave an overall effect of *p* = 0.15 with a large effect size (1.44). This depicted that the BMI is not significantly improved with aerobic training (Fig. 5).

#### Neck circumference (NC)

The NC outcome was reported in four studies totaling 88 participants in the aerobic exercise group and 93 participants in the control group [43, 45–47]. The studies showed no heterogeneity culminating in I^2^ = 0%. The analysis demonstrated that there was no significant decrease in neck circumference outcome as *p* = 0.38 with a large effect size (0.87). The MD and CI values were calculated to be -0.45 and (-1.46, 0.56) respectively which led to a forest plot favouring the aerobic exercise group but not statistically significant (Fig. 5).

#### Maximal oxygen consumption (VO_2max_)

The mean and SD values for VO_2max_ were available in two studies [44, 45]. There was substantial heterogeneity among the studies (I^2^ = 55%) so a random effects model was followed for analysis. Both the studies reported a positive effect of aerobic exercise on VO_2max_ giving MD of 5.84 and CI (0.55, 11.14). This showed a significant improvement in the experimental group denoted by an overall effect of *p* = 0.03 and a large effect size (2.17). These results lead to a forest plot favouring the aerobic exercise group (Fig. 5).

## Discussion

The present study included eight articles for systematic review, of which seven studies were eligible for meta-analysis [40, 42–47]. The primary finding of the study showed significant improvement in AHI among OSA patients with aerobic exercises. Additionally, other outcomes including ODI, mean SpO_2_, ESS, BMI, and neck circumference showed a non-significant improvement. Furthermore, there was a significant increase in the maximal oxygen consumption among the patients who underwent aerobic training [44, 45].

Among the included articles, three studies involved only male participants [40, 41, 46]. However in other studies number of male participants exceeded females except one where the percentage of female participants was more [42]. This could be imputable to the high prevalence of OSA among men as compared to women because of the hormonal differences and fat distribution in the body [48]. Moreover, the mean age of participants at baseline in all the studies was in the range of 45 to 65 years. The increased prevalence of OSA among the middle-aged and elderly population is reflected by this demographic [48]. The use of CPAP by the participants should also be considered as a crucial factor influencing the outcomes of the studies. Among the involved studies, the use of CPAP was followed and the average usage was reported for all the participants in two studies [40, 41]. Karlsen et al. [44] mentioned the use of CPAP by the participants, but the number of hours of machine use was not reported. The CPAP-using individuals were excluded in three studies [43, 45, 47] and two studies did not mention about its use among the participants [42, 46].

The AHI was significantly less in the patients who underwent aerobic training. The baseline AHI of the participants appeared in the category of moderate to severe OSA. The average improvement in the AHI ranged from approximately 4 events/ hour [47] to 21 events/ hour [41]. When the participants were divided into moderate and severe OSA groups for subgroup analysis by Jurado Garcia et al. [43], the reduction in AHI was observed to be 15 events/ hour in the severe OSA group. This can be attributed to a relatively high frequency and longer duration of aerobic training of five days/ week for six months. Thus the implementation of the principles of exercise training (frequency, intensity, duration, and type of exercise) in the intervention group could play an important role in the recovery of OSA patients. However, the significance and analysis of each parameter of the training protocol for these patients is beyond the scope of this review.

The effect of aerobic exercise training on the severity of OSA could be explained with several potential mechanisms. White LH et al. [49] described the role of overnight rostral fluid shift in increasing AHI among OSA patients. Thus aerobic exercises could improve venous blood flow and decrease leg edema in the patients during the day which could further prevent the fluid shift towards the neck at night [50]. Furthermore, Netzer and co-workers [51] described that improved tone of pharyngeal muscles post-physical activity reduces the severity of OSA in the absence of any change in the body weight of the individuals. As demonstrated by Vincent et al. [52] endurance training leads to metabolic adaptations in upper airway muscles that involve an increase in the oxidative capacity and antioxidant enzyme activity with decreased lipid peroxidation, thus enhancing the tone of those muscles.

The current review showed improvement in other OSA indices including ODI, Mean SpO_2_, and ESS, but was not statistically significant. The meta-analysis of ODI and ESS showed minimal improvement in the exercise group with two studies [43, 47] and [43, 46] included respectively, due to the unavailability of data in the remaining studies. Also, the anthropometric measures including BMI and neck circumference showed no statistical difference between aerobic exercise and control groups of the studies. The trials demonstrated contrasting results with the majority of the studies reporting no statistical changes in these outcomes. However, Carneiro-Barrera et al. [41] and Jurado Garcia et al. [43] reported significant reductions in these anthropometric parameters. The redistribution of fluid in pharyngeal walls following long-term aerobic training [43] and loss of body weight with lifestyle changes [41] were the implied explanations for these results. Increased BMI is associated with increase in the apnea and hypopnea episodes, thereby mounting the severity of OSA [53]. However, due to the limited sample size of the studies included in the current meta-analysis of BMI, it is challenging to draw firm conclusions on effects of aerobic training on this outcome.

Aerobic exercise training has a contributed significantly on decreasing the severity and improving other outcome parameters of OSA, but it should not be applied as a single therapy for treatment of patients with severe OSA. It should be accompanied with CPAP and diet modifications to achieve greater improvements in AHI.

### Limitations

There were a few drawbacks in this study. Firstly, a limited number of articles were available for the review and meta-analysis. Secondly, the articles published in languages other than English and unpublished articles were not included. This could account for language and publication bias. Also, the data available for the meta-analysis of the secondary endpoints involving mean SpO_2_, oxygen desaturation during sleep, daytime sleepiness, anthropometric parameters, and VO_2max_ was inadequate and despite repeated attempts to contact the study authors, the required data could not be retrieved.

### Future implications

Future studies should address the other sleep parameters such as snoring, sleep quality, AHI with positional variations, REM and NREM sleep; and exercise parameters such as maximum heart rate and metabolic equivalents to depict the impact of aerobic exercises on OSA patients. The effect of aerobic exercises on objective outcomes of body composition can also be investigated. Further randomized controlled trials could target in comparing the dosage of aerobic exercise training that is frequency, intensity, time, type, volume, and progression of the exercise. The review establishes a need to conduct high-quality RCTs to assess and differentiate the effect of supervised versus unsupervised aerobic training among OSA patients. The training showed positive effects on leptin levels in one study [44], however further RCTs with a larger sample size should be performed. Additionally, trials should be conducted to analyze the effect of aerobic exercises on cognitive functions including attention, memory, and executive functions which appear to be impaired among OSA patients [54].

## Conclusion

The present systematic review and meta-analysis concluded that there is supporting evidence for the beneficial effects of aerobic exercise training in reducing the severity of obstructive sleep apnea. Furthermore, the evidence demonstrated a positive impact on maximal oxygen consumption among OSA patients. The aerobic training showed a non-significant but overall large effect on daytime sleepiness and anthropometric measures.

## Data Availability

Data sharing not applicable.
